# 4D flow MRI in abdominal vessels: prospective comparison of k-t accelerated free breathing acquisition to standard respiratory navigator gated acquisition

**DOI:** 10.1038/s41598-022-23864-9

**Published:** 2022-11-18

**Authors:** Octavia Bane, Daniel Stocker, Paul Kennedy, Stefanie J. Hectors, Emilie Bollache, Susanne Schnell, Thomas Schiano, Swan Thung, Aaron Fischman, Michael Markl, Bachir Taouli

**Affiliations:** 1 Department of Diagnostic, Molecular and Interventional Radiology, Icahn School of Medicine at Mount Sinai, 1470 Madison Avenue, New York, NY 10029 USA; 2 BioMedical Engineering and Imaging Institute, Icahn School of Medicine at Mount Sinai, New York, NY USA; 3 Department of Biomedical Engineering, McCormick School of Engineering, Northwestern University, Evanston, IL USA; 4 Laboratoire d’Imagerie Biomédicale, INSERM, Paris, France; 5 Department of Medical Physics, Universität Greifswald, Greifswald, Germany; 6 Recanati/Miller Transplantation Institute, Icahn School of Medicine at Mount Sinai, New York, NY USA; 7 Department of Pathology, Icahn School of Medicine at Mount Sinai, New York, NY USA; 8 Department of Radiology, Feinberg School of Medicine, Northwestern University, Chicago, IL USA

**Keywords:** Biomedical engineering, Portal hypertension, Translational research

## Abstract

Volumetric phase-contrast magnetic resonance imaging with three-dimensional velocity encoding (4D flow MRI) has shown utility as a non-invasive tool to examine altered blood flow in chronic liver disease. Novel 4D flow MRI pulse sequences with spatio-temporal acceleration can mitigate the long acquisition times of standard 4D flow MRI, which are an impediment to clinical adoption. The purpose of our study was to demonstrate feasibility of a free-breathing, spatio-temporal (k−t) accelerated 4D flow MRI acquisition for flow quantification in abdominal vessels and to compare its image quality, flow quantification and inter-observer reproducibility with a standard respiratory navigator-gated 4D flow MRI acquisition. Ten prospectively enrolled patients (M/F: 7/3, mean age = 58y) with suspected portal hypertension underwent both 4D flow MRI acquisitions. The k−t accelerated acquisition was approximately three times faster (3:11 min ± 0:12 min/9:17 min ± 1:41 min, *p* < 0.001) than the standard respiratory-triggered acquisition. Vessel identification agreement was substantial between acquisitions and observers. Average flow had substantial inter-sequence agreement in the portal vein and aorta (CV < 15%) and poorer agreement in hepatic and splenic arteries (CV = 11–38%). The k−t accelerated acquisition recorded reduced velocities in small arteries and reduced splenic vein flow. Respiratory gating combined with increased acceleration and spatial resolution are needed to improve flow measurements in these vessels.

## Introduction

4D flow magnetic resonance imaging (MRI) using phase contrast pulse sequences with 3D vascular coverage and 3-direction velocity encoding allows volumetric visualization and quantification of blood flow, from a single acquisition^1–5^. 4D flow MRI has emerged as a non-invasive tool for assessment of altered hemodynamics in chronic liver disease^3,6^ and portal hypertension (PH)^7–11^, for visualization of stenosis and collateral circulation^12^, and surgical planning for liver transplantation^12^. 4D flow MRI measurements of splanchnic hemodynamics have been correlated with an imaging-based score of portal hypertension^7^, and have been shown to predict risk of bleeding from gastroesophageal varices in patients with portal hypertension^13^. Despite the potential utility of 4D flow MRI as a non-invasive tool to assess complications from advanced liver disease, its lengthy acquisition times have impeded the clinical adoption of 4D flow MRI. A free-breathing, shorter acquisition would increase the clinical utilization of 4D flow MRI by facilitating patient compliance, and better integration into liver MRI protocols. The need for respiratory control to reduce blurring and ghosting artifacts from breathing motion in abdominal applications further increases acquisition times. Despite the use of advanced respiratory navigator techniques^14^, parallel imaging, or radial sampling k-space patterns^8,9,15^, 4D flow MRI with respiratory gating has a data acquisition efficiency of 60–80%, and acquisition times of 10–20 min^6^.

Several 4D flow MRI studies have investigated advanced acceleration techniques to decrease acquisition time. Spiral sampling combined with compressed sensing allowed the acquisition of 4D flow MRI data covering the major abdominal vessels in a single breath-hold (20–24 s)^5,7^. A free-breathing acquisition using spatio-temporal (k−t) parallel imaging with extended and averaged GRAPPA (Generalized Autocalibrating Partial Parallel Acquisition) kernels (PEAK GRAPPA)^16,17^, has been shown to achieve equivalent image quality and flow quantification in the aorta as a conventional 4D flow MRI, navigator-gated Cartesian sequence, in under 2 min^18^. The use of advanced acceleration techniques in combination with free breathing has not previously been examined to quantify flow in the abdominal circulation of patients with liver disease.

Hence, the objective of our study was to demonstrate the feasibility of a free-breathing, k−t (3D k-space and time) accelerated 4D flow MRI acquisition for flow quantification in abdominal vessels with reduced acquisition time and to compare with a standard respiratory navigator-gated 4D flow MRI acquisition in terms of image quality, flow quantification and interobserver reproducibility of measurements.

## Results

### Patients

Between March and July 2018, we enrolled ten initial adult patients (M/F: 7/3, mean age 58 years, range 37–73 years) with chronic liver disease, with suspected portal hypertension (PH), no prior/concomitant pharmacologic treatment for PH, no portal vein thrombosis (as determined based on prior clinical imaging) and no contraindications for MRI. The etiologies of liver disease were: chronic hepatitis C viral infection (n = 2), non-alcoholic fatty liver disease/steatohepatitis (n = 5), primary sclerosing cholangitis (n = 1), alcohol abuse (n = 1), and unknown (n = 1). Seven of the ten patients had transjugular liver biopsy with hepatic venous pressure gradient (HVPG) measurements within up to one month (10.5 ± 11.5 days) of MRI.

### Acquisition time and image quality

**Figure 1:**
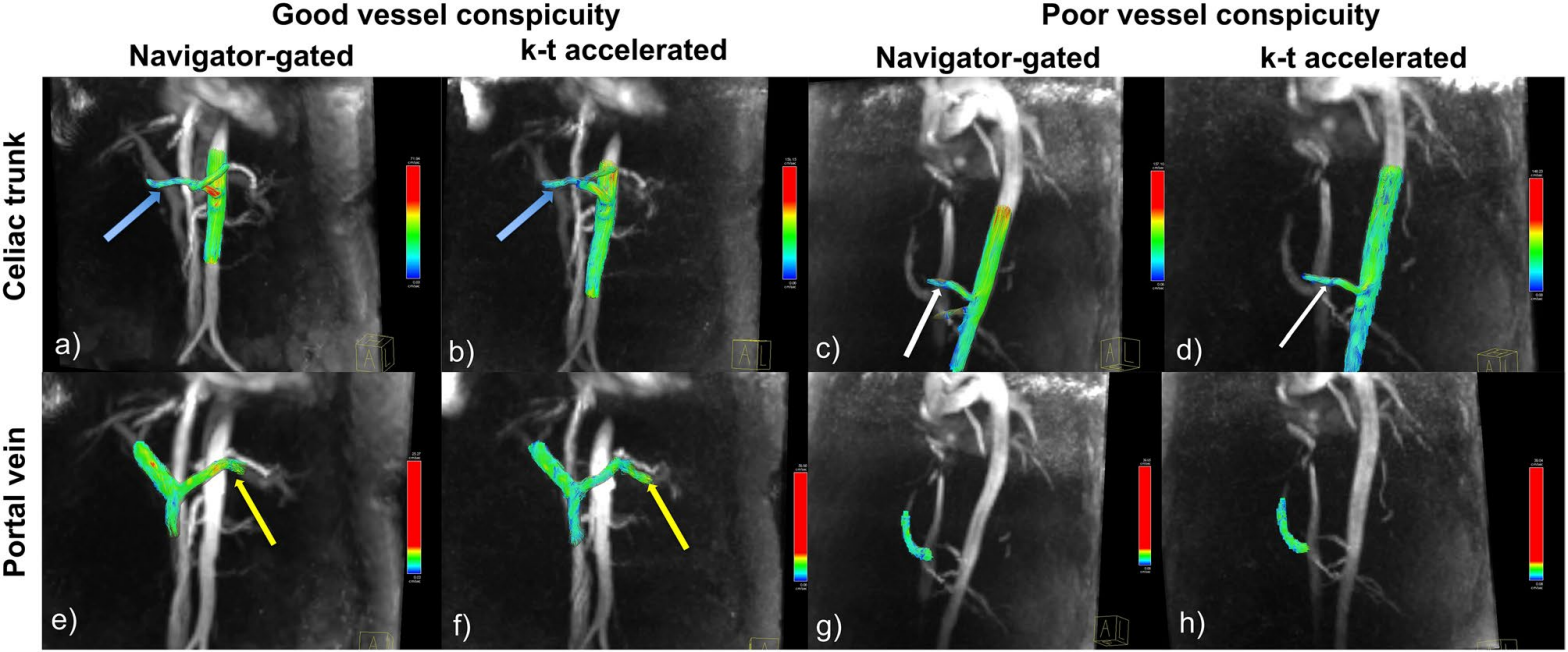
Example of good vessel conspicuity on both the navigator-gated and k−t accelerated sequence, in a 64 year-old female with HVPG = 14 mmHg (**a**, **b**, **e**, **f**), and of poor vessel conspicuity, in a male, 57 year-old patient with HVPG = 17 mmHg (**c**, **d**, **g**, **h**). The hepatic artery (blue arrow; **a**, **b**) had a vessel conspicuity score of 2 on both acquisitions, while the the supraceliac aorta, celiac trunk, splenic artery (**a**, **b**), portal vein, SMV (**e**, **f**) all had a vessel conspicuity score of 3 on both acquisitions. The SV (yellow arrow) had a score of 3 on the navigator-gated sequence (**e**), and a score of 2 on the k−t accelerated sequence (**f**). In the case with poor vessel conspicuity, the hepatic artery (white arrow; **c**–**d**) had a score of 1 on both acquisitions, the supraceliac aorta, celiac trunk, hepatic artery (**c**, **d**) and portal vein (**g**, **h**) had a score of 3 on both acquisitions, while the splenic artery, SMV, and SV (**g**, **h**) were scored 0 (not seen) on both acquisitions. Background noise and artifacts were scored as 3 (minimal to none) for both cases with both acquisitions.

The free-breathing, k−t accelerated sequence achieved approximately a three-fold reduction in acquisition time compared to the navigator-gated Cartesian sequence (3:11 ± 0:12 min vs. 9:17 ± 1:41 min, *p* < 0.001). For both observers, conspicuity of the aorta, celiac trunk, portal vein, middle hepatic vein (all *p* > 0.102) and background artifact scores (p = 0.564) were equivalent between the two sequences (Table 1). However, conspicuity scores for both observers in the hepatic artery and the splenic vein were significantly lower with the k−t accelerated sequence (Fig. 1), compared to the navigator-gated sequence (all *p* < 0.038). Portal vein flow was hepatopetal in all patients with both acquisitions.

**Table 1:** Qualitative scoring of vessel conspicuity and background artifacts with the two acquisitions, as evaluated by two observers.

	Aorta	Celiac trunk	HA	SA	PV	SMV	SV	MHV	Background Artifacts
**Observer 1**									
k−t accelerated, no respiratory gating	3 (0)	3 (0)	2 (1.5)	2.5 (1)	3 (0)	3 (1.5)	1.5 (1.5)	2 (1)	2.5 (1)
Navigator-gated	3 (0)	3 (0)	3 (1)	2.5 (1)	3 (0)	3 (0)	3 (0.5)	2.5 (2)	2 (0.5)
p*	> 0.99	> 0.99	**0.034**	0.564	0.18	0.109	**0.015**	**0.046**	0.157
**Observer 2**									
k−t accelerated, no respiratory gating	3 (0)	3 (0)	1 (1)	2 (1)	2.5 (1)	2 (1)	1 (1)	1.5 (2)	3 (1)
Navigator-gated	3 (0)	3 (0)	2 (2)	3 (0)	3 (0)	3 (1)	2.5 (1)	2 (1)	2.5 (1)
p*	> 0.99	> 0.99	**0.038**	**0.034**	0.102	**0.035**	**0.009**	0.257	0.564

Data is shown as median (IQR) of the vessel conspicuity (0: vessel not seen; 1: severely to moderately blurred; 2: mildly blurred; and 3: well delineated) and background artifacts scores (1: severe artifacts; 2: moderate; 3: minimal or none). Vessel conspicuity scores for the two acquisitions were equivalent in large vessels, but significantly lower for the k−t accelerated acquisition in the hepatic artery and splenic vein (*p* < 0.05 is shown in bold font). Significant differences on paired Wilcoxon test (p* < 0.05) are shown in bold font. *HA*  hepatic artery, *MHV*   middle hepatic vein, *PV*  portal vein, *SA*  splenic artery, *SMV*   superior mesenteric vein, *SV*   splenic vein.

### Inter-observer agreement in vessel identification and flow quantification

Inter-observer agreement (Table 2) for vessel identification (kappa = 0.63–1) was substantial to excellent with both acquisitions. In all vessels, hemodynamic measurements with both sequences showed acceptable inter-observer agreement with CV < 20%, Bland–Altman bias < 20%, but high Bland–Altman limits of agreement (BALA) within [−60%, 60%]. Interobserver agreement of hemodynamic measurements with both sequences was substantial for the abdominal aorta (Table 2; CV < 15%) and portal vein (CV < 20%). Poor interobserver agreement was observed with the k−t acquisition in the less conspicuous splenic vein (Table 2; CV = 26.8%).

**Table 2:** Inter-observer agreement in vessel identification and flow quantification with the free breathing k−t accelerated and the standard Cartesian navigator-gated acquisition.

		All vessels		Arteries	Veins	Aorta	Celiac trunk	HA	SA	PV	SMV	SV	MHV
**Free-breathing, k−t accelerated acquisition**													
	Cohen's kappa	0.945		1	0.918	1	1	1	1	1	1	1	0.737
	*p*	< 0.001		< 0.001	< 0.001	< 0.001	< 0.001	0.002	0.002	< 0.001	0.002	0.002	0.016
Average Velocity (cm/s)	CV (%)	9.9		10.3	9.4	2.6	8.7	13.3	18.0	8.7	4.5	20.1	5.1
	Bias (%)	1.2		−0.3	2.9	0.8	−2.7	6.3	−4.7	−7.2	5.1	15.5	2.3
	BALA (%)	−41.8,44.2		−47.3,46.8	−35.4, 41.3	−8.8,10.4	−32.9,27.6	−37.2,49.8	−87.7,78.3	−36.6,22.3	−8.5,18.6	−50.8,81.9	−15.0,19.5
Maximum Velocity (cm/s)	CV (%)	13.4		13.6	13.2	3.6	13.5	18.1	20.6	16.5	7.7	18.5	9.5
	Bias (%)	−0.6		0.8	−2.1	−0.3	−0.2	−0.1	3.9	−12.2	4.8	−5.2	7.7
	BALA (%)	−50.6,49.5		−52.1,53.6	−49.4, 45.3	−13.7,13.1	−52.3,51.8	−70.1,69.9	−66.6,74.5	−66.3,41.9	−24.4,34.0	−67.8,57.4	−22.5,38.0
Flow (ml/s)	CV (%)	13.1		12.9	13.3	5.0	11.4	17.6	19.0	5.6	13.0	26.8	11.1
	Bias (%)	−1.4		−3.8	1.4	6.3	−1.6	−1.1	−19.9	−4.3	6.5	10.9	−5.9
	BALA (%)	−56.6,53.8		−62.9, 55.3	−49.3,52.0	−15.4,28.0	−50.9,47.7	−69.1,67.0	−102.0,62.3	−20.1,11.5	−45.0,58.0	−76.5,98.3	−40.2, 28.3
Area (mm^2^)	CV (%)	10		9.5	10.5	4.3	8.0	14.4	12.7	8.3	10.0	9.4	15.4
	Bias (%)	−2.7		−4.0	−1.2	4.1	1.0	−7.4	−15.6	3.6	1.6	−4.3	−7.9
	BALA (%)	−40.8,35.4		−41.1, 33.1	−40.7,38.4	−15.6,23.8	−33.4,35.4	−59.7,44.8	−46.8,15.6	−30.0,37.1	−41.7.44.8	−41.1,32.4	−57.2,41.5
**Cartesian navigator-gated acquisition**													
	Cohen's kappa		0.820	0.655	0.875	1	1	0.759	1	1	0.632	1	1
	*p*		< 0.001	< 0.001	< 0.001	< 0.001	< 0.001	0.003	< 0.001	< 0.001	0.011	< 0.001	< 0.001
Average Velocity (cm/s)	CV (%)		10.2	15.1	6.1	3.8	16.3	19.6	18.3	4.4	5.1	4.8	9.9
	Bias (%)		3.9	7.6	−1.1	4.0	21.9	12.3	−5.6	−0.1	−5.2	2.7	1.8
	BALA (%)		−43.3, 51.0	−54.4, 69.6	−25.7, 23.6	−15.7,23.8	−27.0,70.7	−66.7,91.2	−82.2,71.0	−17.2,17.0	−19.4,9.0	−17.0,22.4	−35.7,39.2
Maximum Velocity (cm/s)	CV (%)		13.7	17.1	9.3	10.6	20.3	20.8	19.7	7.3	6.0	8.4	16.9
	Bias (%)		2.4	4.9	−2.5	−1.9	23.4	8.0	−11.4	2.5	−2.9	9.3	−5.7
	BALA (%)		−51.5,56.2	−61,70.8	−41.5, 36.6	−44.1,40.4	−35.0,81.8	−70.6,86.5	−80.2,57.3	−22.7,27.7	−24.5,18.8	−13.9,32.6	−69.1, 57.8
Flow (ml/s)	CV (%)		11.3	16.3	6.1	4.0	20.0	18.8	20.0	3.2	7.1	4.4	15.3
	Bias (%)		1.3	4.4	−1.73	4.6	13.0	10.3	−11.4	2.6	−4.3	1.2	−4.4
	BALA (%)		−47.6,50.1	−60.0, 68.7	−31.7, 28.2	−16.9,26.1	−56.9,83.0	−53.7,74.4	−86.2,63.3	−8.4,13.5	−33.9,25.2	−20.0,22.3	−57.2, 48.4
Area (mm^2^)	CV (%)		9	10.4	8.6	1.9	16.6	11.0	10.9	5.0	6.2	3.7	16.9
	Bias (%)		−3.7	−5.7	−0.6	0.8	−16.6	−2.7	−5.9	2.7	0.8	−1.6	−6.6
	BALA (%)		−44.0,36.6	−53.9,42.7	−38.4, 37.2	−5.6,7.2	−85.0,51.7	−41.6, 36.3	−46.5,34.7	−16.6,21.9	−25.3, 27.0	−15.6, 12.5	−66.5, 53.3

Inter-observer agreement in vessel identification is measured by Cohen’s kappa. Inter-observer agreement of hemodynamic parameters is measured by inter-patient mean coefficient of variation (mean CV (%) = 100 SD/mean), Bland–Altman bias, and Bland–Altman limits of agreement (BALA). *HA* hepatic artery, *MHV*   middle hepatic vein, *PV*   portal vein, *SA*  splenic artery, *SMV*  superior mesenteric vein, *SV*   splenic vein.

### Inter-acquisition differences in vessel identification and flow quantification

**Figure 2:**
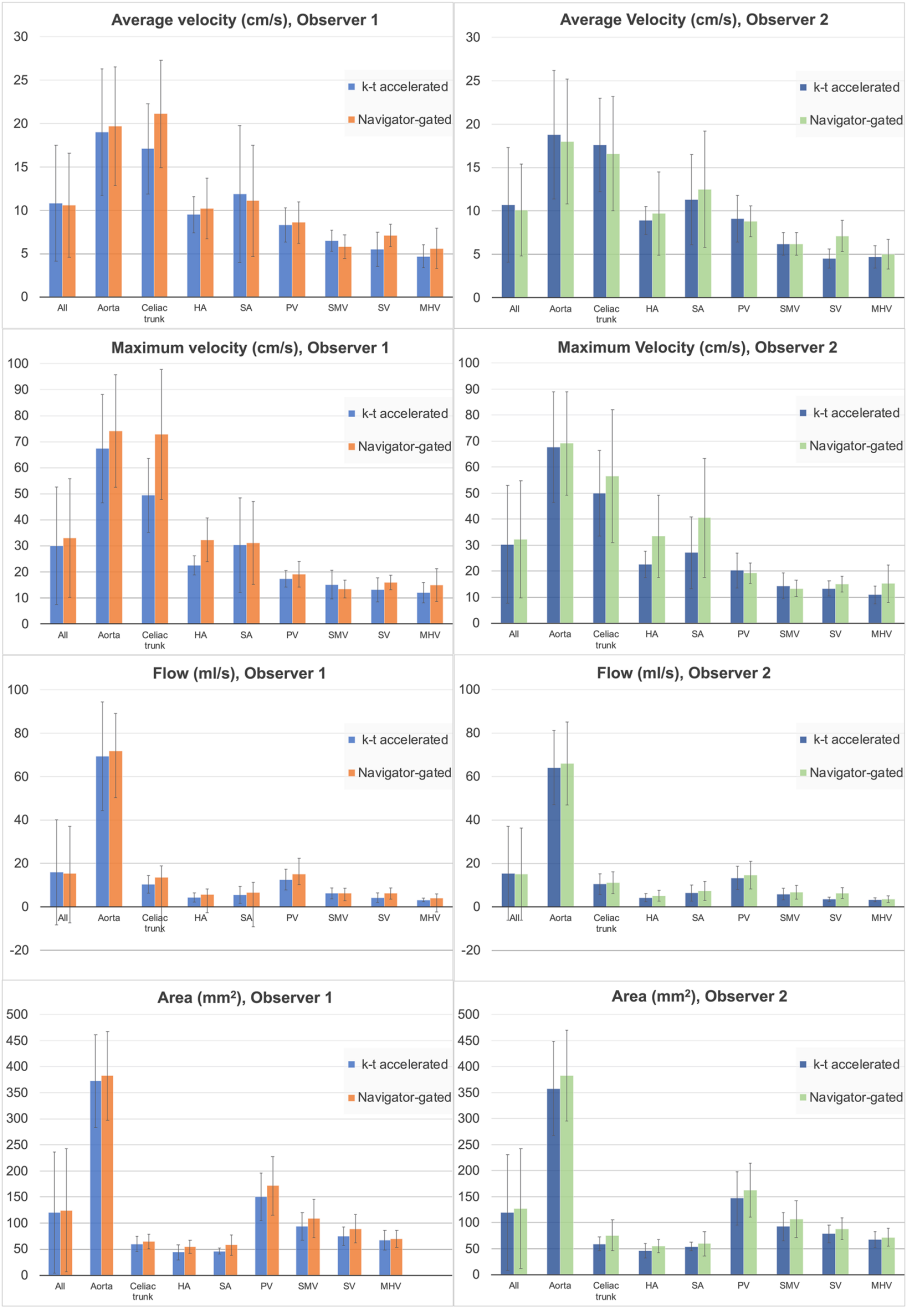
Hemodynamic parameters measured by both observers with both acquisitions, in all vessels. Bar graphs show mean ± standard deviation. *HA*  hepatic artery, *MHV*   middle hepatic vein, *PV*   portal vein, *SA*  splenic artery, *SMV*   superior mesenteric vein, *SV*  splenic vein.

Flow parameters measured by both observers with both acquisitions in all vessels of interest are shown in Fig. 2 and Tables 3, 4. Agreement in vessel identification between measurements with the k−t and the navigator-gated acquisition by both observers (Tables 3, 4) was substantial to excellent (kappa 0.6–1), except for moderate agreement in the hepatic artery and the middle hepatic vein (kappa < 0.5). In the abdominal aorta, agreement between the two acquisitions was excellent for velocity and area measurements (Tables 3, 4; for both observers: CV = 4.5–9.3%, bias < 10%, BALA within [−42%, 33%]), and acceptable for flow (CV < 17%, bias < 12%, BALA within [−56.1%, 78.2%]). In the portal vein, agreement in hemodynamic measurements was acceptable (CV < 20%, bias = 0.6–10%, BALA within [−42%, 62%]). Agreement in hemodynamic measurements for small vessels such as the hepatic and splenic arteries, was modest, with CV = 11–38%, bias = 10–40%, and BALA within [−101%, 103%].

**Table 3:** Inter-sequence agreement in vessel identification and hemodynamic parameters measured by observer 1 on the free breathing k−t accelerated and the standard Cartesian navigator-gated acquisitions in abdominal vessels, along with intersequence differences in parameters.

Observer 1										
		All vessels	Aorta	Celiac trunk	HA	SA	PV	SMV	SV	MHV
	Cohen's kappa	0.74	1	1	0.42	1	1	1	0.792	0.744
	*p*	< 0.001	0.001	0.001	0.087	0.001	0.001	0.001	0.001	0.11
Average Velocity (cm/s)	Free-breathing k−t accelerated acquisition	10.8 ± 6.7	19.0 ± 7.3	17.1 ± 5.2	9.5 ± 2.1	11.9 ± 7.9	8.3 ± 2.0	6.5 ± 1.2	5.5 ± 2.0	4.7 ± 1.3
	Navigator−gated acquisition	10.6 ± 6.0	19.7 ± 6.8	21.1 ± 6.2	10.2 ± 3.5	11.1 ± 6.4	8.6 ± 2.4	5.8 ± 1.4	7.1 ± 1.3	5.6 ± 2.3
	Difference (%)	0.18 ± 28.7	8.1 ± 11.4	−4.5 ± 31.2	−1.6 ± 19.3	12.8 ± 54.1	−1.9 ± 18.9	8.5 ± 22.4	−21.7 ± 26.5	−2.9 ± 23.3
	*p*	0.518	0.131	0.375	0.313	0.652	0.625	0.383	0.109	0.313
	CV (%)	14.9	7.4	17.0	12.7	25.5	10.7	11.2	24.6	12.7
	Bias (%)	3.8	−7.3	9.7	3.3	−1.0	3.5	−6.3	28.6	5.3
	BALA (%)	−53.9,61.6	−28.0,13.5	−61.9,81.3	−35.4,42.0	−100.8,98.8	−33.0,40.0	−45.8,33.2	−38.0,95.3	−39.2,49.7
Maximum Velocity (cm/s)	Free-breathing k−t accelerated acquisition	30.0 ± 22.6	67.4 ± 20.8	49.4 ± 14.2	22.5 ± 3.7	30.3 ± 18.2	17.3 ± 3.2	15.1 ± 5.5	13.1 ± 4.6	12.0 ± 3.9
	Navigator-gated acquisition	33.0 ± 22.8	74.1 ± 21.6	72.8 ± 25.0	32.3 ± 8.4	31.1 ± 15.9	19.1 ± 4.9	13.4 ± 3.4	15.9 ± 2.8	14.9 ± 6.3
	Difference (%)	**−8.4 ± 31.8**	6.9 ± 22.1	**−20.2 ± 21.9**	**−31.3 ± 16.2**	−4.7 ± 50.9	−6.4 ± 28.1	16.0 ± 37.8	−17.6 ± 24.4	−12.8 ± 24.3
	*p*	** < 0.001**	0.922	**0.010**	**0.008**	0.820	0.193	0.461	0.219	0.109
	CV (%)	18.8	9.3	20.6	27.6	29.1	15.3	14.9	18.0	17.3
	Bias (%)	13.8	−4.8	25.6	39.0	14.9	10.0	−10.6	23.0	16.7
	BALA (%)	−48.2,75.7	−41.8,32.2	−30.9,82.0	−5.1,83.1	−72.7,102.6	−41.8, 61.7	−65.4,44.3	−41.2,87.2	−36.1,69.6
Flow (ml/s)	Free-breathing k−t accelerated acquisition	15.9 ± 24.2	69.4 ± 25.0	10.4 ± 4.1	4.3 ± 2.1	5.5 ± 4.0	12.5 ± 4.8	6.2 ± 2.5	4.2 ± 2.2	3.1 ± 0.8
	Navigator-gated acquisition	15.4 ± 21.6	71.9 ± 17.2	13.5 ± 5.3	5.7 ± 2.6	6.6 ± 4.7	15.1 ± 7.2	6.2 ± 2.4	6.3 ± 2.4	3.9 ± 2.0
	Difference (%)	−5.5 ± 28.8	6.3 ± 12.9	−6.5 ± 27.6	−17.1 ± 30.1	0.7 ± 42.2	−7.7 ± 23.2	2.7 ± 27.6	−**29.7 ± 17.0**	1.1 ± 34.5
	*p*	0.123	0.375	0.432	0.250	1.000	0.432	0.844	**0.016**	0.547
	CV (%)	6.6	16.7	24.3	23.7	14.7	15.7	26.1	21.4	6.6
	Bias (%)	−5.5	11.0	24.2	7.4	10.8	0.6	36.9	4.1	−5.5
	BALA (%)	−28.6,17.7	−56.1,78.2	−52.3,100.7	−79.6,94.4	−41.2,62.9	−53.6,54.7	−9.4,83.3	−63.4,71.5	−28.6,17.7
Area (mm^2^)	Free-breathing k−t accelerated acquisition	120.2 ± 115.6	372.3 ± 89.2	59.8 ± 14.5	44.0 ± 14.9	46.2 ± 5.8	151.0 ± 45.5	93.8 ± 26.9	75.0 ± 17.4	67.3 ± 18.8
	Navigator-gated acquisition	124.2 ± 118.3	382.3 ± 85.7	64.8 ± 13.6	54.4 ± 12.5	57.7 ± 19.7	171.6 ± 56.4	109.0 ± 36.8	89.1 ± 27.4	69.8 ± 16.8
	Difference (%)	**−4.8 ± 19.3**	−4.1 ± 5.9	−0.7 ± 13.4	−16.2 ± 23.8	−6.2 ± 19.0	−5.5 ± 15.9	−5.6 ± 15.7	−7.2 ± 12.8	6.1 ± 37.2
	*p*	**0.0023**	0.084	0.922	0.195	0.426	0.275	0.461	0.156	0.844
	CV (%)	10.4	4.5	7.6	18.0	11.3	10.1	9.6	8.4	15.7
	Bias (%)	6.8	4.3	1.5	21.3	8.4	6.9	7.0	8.2	−1.3
	BALA (%)	−32.6,46.2	−7.6,16.2	−24.6,27.5	−41.8,84.4	−36.6,53.3	−25.9,39.7	−27.3,41.3	−18.8,35.2	−59.2,56.7

Agreement in vessel identification between acquisitions is measured by Cohen’s kappa. Mean and standard deviation of hemodynamic measurements with each acquisition are given for all vessels and for each individual vessel. Significant differences (paired Wilcoxon *p* < 0.05) in hemodynamic acquisitions are shown in bold font. Percent difference between the k−t no respiratory gating and the navigator-gated acquisition was calculated in vessels identified by both sequences as: Difference (%) = 100 x (k−t Measurement-Gated Measurement)/ Gated Measurement. Intersequence agreement of hemodynamic parameters is measured by inter-patient mean coefficient of variation (mean CV (%) = 100 SD/mean), Bland–Altman bias, and Bland–Altman limits of agreement (BALA). *HA*  hepatic artery, *MHV*   middle hepatic vein, *PV*  portal vein, *SA * splenic artery, *SMV*   superior mesenteric vein, *SV*   splenic vein.

**Table 4:** Inter-sequence agreement in vessel identification and hemodynamic parameters measured by observer 2 on the free breathing k−t accelerated and the standard Cartesian navigator-gated acquisitions in abdominal vessels, along with intersequence differences in parameters.

Observer 2										
		All vessels	Aorta	Celiac trunk	HA	SA	PV	SMV	SV	MHV
	Cohen's kappa	0.674	1	1	0.615	1	1	0.615	0.737	0.412
	*p*	< 0.001	0.001	0.001	0.035	0.001	0.001	0.001	0.016	0.107
Average Velocity (cm/s)	Free-breathing k−t accelerated acquisition	10.7 ± 6.6	18.8 ± 7.4	17.6 ± 5.4	8.9 ± 1.6	11.3 ± 5.2	9.1 ± 2.7	6.2 ± 1.3	4.5 ± 1.1	4.7 ± 1.3
	Navigator-gated acquisition	10.1 ± 5.3	17 ± 6.4	14.0 ± 5.0	9.7 ± 4.1	11.0 ± 3.8	8.7 ± 1.8	6.6 ± 1.1	7.1 ± 1.8	4.9 ± 2.1
	Difference (%)	4.1 ± 34.9	**10.5 ± 8.1**	29.7 ± 37.6	2.1 ± 51.8	7.7 ± 47.1	3.4 ± 18.3	−5.1 ± 14.1	**−34.3 ± 21.3**	5.6 ± 35.6
	*p*	0.717	**0.006**	0.064	0.313	0.496	0.846	0.461	**0.031**	0.469
	CV (%)	16.5	7.4	22.5	19.8	18.4	9.7	8.3	32.6	17.7
	Bias (%)	1.3	−9.7	−21.3	7.0	0.7	−2.0	6.2	44.6	−0.8
	BALA (%)	−62.2,64.8	−24.0,4.6	−82.4,39.8	−74.8,88.8	−82.9,84.2	−35.0,31.1	−24.2,36.6	−14.0,103.2	−61.8,60.1
Maximum Velocity (cm/s)	Free-breathing k−t accelerated acquisition	30.3 ± 22.6	67.6 ± 21.2	50.0 ± 16.4	22.7 ± 5.1	27.1 ± 13.7	20.3 ± 6.7	14.4 ± 4.9	13.4 ± 2.8	10.9 ± 3.5
	Navigator-gated acquisition	32.3 ± 22.5	68.7 ± 19.7	47.7 ± 20.3	34.9 ± 16.1	35.8 ± 15.1	19.6 ± 4.0	14.1 ± 3.0	15.0 ± 3.2	16.3 ± 8.5
	Difference (%)	−4.9 ± 30.5	−1.8 ± 8.3	8.7 ± 18.2	−16.3 ± 53.4	−19.1 ± 29.2	5.0 ± 30.5	3.7 ± 27.8	−5.1 ± 38.6	−21.0 ± 18.6
	*p*	0.064	0.557	0.375	0.148	0.074	0.695	0.742	0.375	0.078
	CV (%)	17.4	4.4	11.3	37.4	22.8	14.8	14.0	20.2	19.3
	Bias (%)	8.1	2.1	−7.0	31.6	27.2	−0.9	−0.5	11.1	25.5
	BALA (%)	−56.7,76.8	−14.9,19.2	−41.1,27.1	−80.5,143.8	−58.2,112.5	−60.1,58.3	−53.4, 52.5	−57.7,79.9	−18.9,69.8
Flow (ml/s)	Free-breathing k−t accelerated acquisition	15.4 ± 21.6	64.1 ± 17.0	10.5 ± 4.8	4.2 ± 1.9	6.4 ± 3.7	13.3 ± 5.4	5.9 ± 2.6	3.5 ± 1.0	3.2 ± 0.9
	Navigator-gated acquisition	15.1 ± 21.2	63.7 ± 21.0	9.7 ± 4.6	5.1 ± 2.5	5.7 ± 2.0	13.9 ± 6.9	7.3 ± 3.6	6.0 ± 2.2	3.5 ± 1.7
	Difference (%)	−1.4 ± 37.7	2.9 ± 10.5	16.7 ± 48.0	−9.5 ± 41.6	12.6 ± 52.5	−1.1 ± 25.1	−7.4 ± 34.8	**−36.6 ± 26.8**	−0.1 ± 35.0
	*p*	0.197	0.770	0.695	0.383	0.734	0.846	0.313	**0.031**	0.375
	CV (%)	20.6	5.4	25.1	25.4	20.9	14.4	22.1	38.4	19.6
	Bias (%)	10.0	−2.4	−7.0	18.7	−2.9	4.0	13.5	49.8	5.2
	BALA (%)	−65.4,81.7	−21.8,17.0	−91.4,77.4	−73.7,111.1	−86.8,81.0	−46.2,54.1	−58.5,85.4	−20.0,119.6	−62.2,72.6
Area (mm^2^)	Free-breathing k−t accelerated acquisition	119.8 ± 111.0	357.6 ± 90.7	58.9 ± 13.3	46.6 ± 14.0	54.3 ± 8.3	147.1 ± 51.3	92.6 ± 27.4	78.4 ± 17.3	68.1 ± 15.2
	Navigator-gated acquisition	127.3 ± 115.5	386.3 ± 89.5	79.1 ± 33.3	52.8 ± 8.2	52.3 ± 8.6	154.2 ± 51.7	107.9 ± 41.1	84.3 ± 19.7	70.2 ± 8.0
	Difference (%)	**−5.9 ± 22.2**	**−7.2 ± 9.5**	**−19.8 ± 20.3**	−9.3 ± 25.2	4.8 ± 14.2	−3.5 ± 24.1	−3.1 ± 33.2	−2.7 ± 28.1	−4.2 ± 18.8
	*p*	** < 0.001**	**0.037**	**0.020**	0.461	0.496	0.375	0.313	0.469	0.688
	CV (%)	13.7	7.6	19.5	18.5	8.1	13.3	16.9	15.6	11.1
	Bias (%)	8.7	8.0	24.7	12.9	−3.8	6.1	7.6	6.0	6.0
	BALA (%)	−37.0, 54.5	−12.1, 28.0	−29.1,78.6	−41.0,66.8	−30.2,22.5	−38.9,51.0	−52.7,68.0	−46.6,58.5	−34.6,46.6

Agreement in vessel identification between acquisitions is measured by Cohen’s kappa. Mean and standard deviation of hemodynamic measurements with each acquisition are given for all vessels and for each individual vessel. Significant differences (paired Wilcoxon *p* < 0.05) in hemodynamic acquisitions are shown in bold font. Percent difference between the k−t no respiratory gating and the navigator-gated acquisition was calculated in vessels identified by both sequences as: Difference (%) = 100 x (k−t Measurement-Gated Measurement)/ Gated Measurement. Intersequence agreement of hemodynamic parameters is measured by inter-patient mean coefficient of variation (mean CV (%) = 100 SD/mean), Bland–Altman bias, and Bland–Altman limits of agreement (BALA). *HA*   hepatic artery;, *MHV*   middle hepatic vein, *PV*   portal vein, *SA*   splenic artery, *SMV*   superior mesenteric vein, *SV*  splenic vein.

For all vessels, maximum velocity measured by observer 1 (Table 3) and cross-section area measured by both observers were lower with the k−t acquisition (Tables 3, 4*p* < 0.003). Maximum velocities in the celiac trunk (Difference = −20.2 ± 21.9%, *p* < 0.001) and hepatic artery (Difference = -31.3 ± 16.2%, *p* = 0.008) measured by observer 1 (Table 3) were significantly lower with the k−t acquisition (*p* < 0.01). For observer 2 (Table 4), area measurements in the celiac trunk and aorta were lower with the k−t acquisition (*p* < 0.04), while average velocity in the aorta was slightly higher (*p* = 0.006), as more fast-flow voxels at the center of the vessel than low-flow voxels at the edges were included. For both observers, flow in the splenic vein (*p* < 0.035) measured with the k−t acquisition was as much as 37% lower than the measurements with the navigator-gated acquisition. For the 2nd observer (Table 4), lower average velocity was also observed in the splenic vein with the k−t sequence (Difference = −34.3 ± 21.3, *p* = 0.031).

### Correlation with HVPG

**Figure 3:**
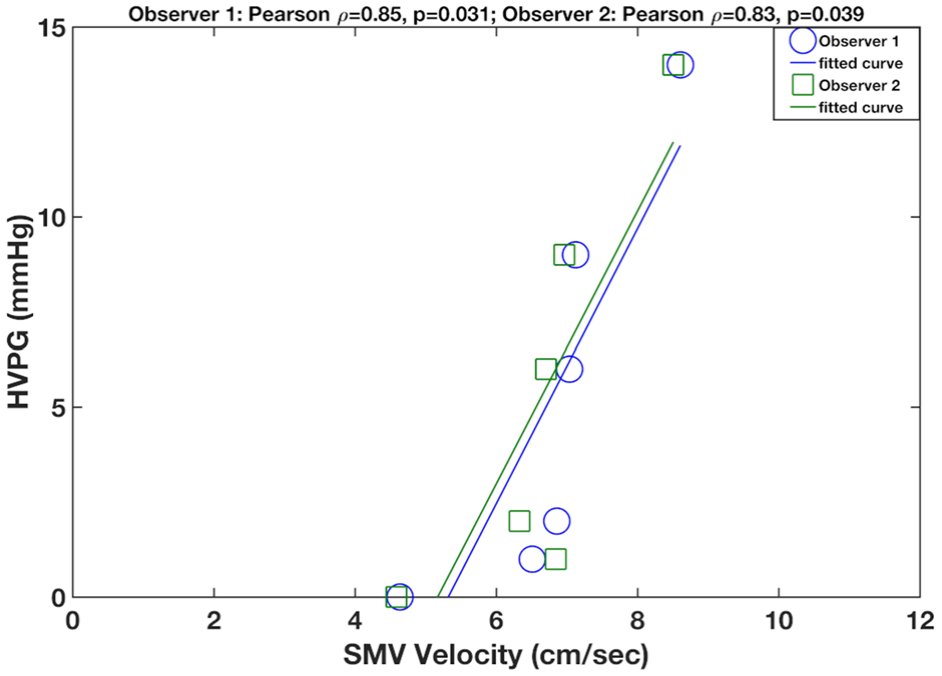
Correlation plots between average velocity in the superior mesenteric vein (SMV) measured with the k−t accelerated acquisition and HVPG measurements in 6 patients with transjugular biopsy. SMV velocity measured by observer 1 (Pearson’s r = 0.85, *p* = 0.031) and observer 2 (Pearson r = 0.83, *p* = 0.039) were strongly correlated to HVPG.

Seven of the ten patients had HVPG measurement, with mean HVPG of 7 ± 6.6 mmHg (range 0–17 mmHg). Average velocity in the superior mesenteric vein measured with the k−t accelerated acquisition was significantly correlated with HVPG (Fig. 3); observer 1/ observer 2**:** Pearson’s r = 0.85, *p* = 0.031/r = 0.83, *p* = 0.039). This correlation was not observed for average velocity measured with the navigator-gated acquisition (observer 1/observer 2: Pearson’s r = 0.68, *p* = 0.137/r = 0.63, *p* = 0.179). No other hemodynamic parameters were significantly correlated to HVPG (*p* = 0.081–0.9).

## Discussion

We sought to assess the feasibility of a k−t PEAK GRAPPA accelerated sequence for 4D flow MRI quantification in the abdominal vasculature without respiratory control. The k−t sequence was compared to a standard navigator-gated Cartesian 4D flow MRI acquisition in 10 patients with liver disease. We found that the k−t accelerated acquisition is feasible in the abdomen and achieves three-fold reduction in acquisition time compared to the navigator-gated 4D flow MRI. However, image quality was significantly lower, inter-observer agreement for flow measurements was modest and there was substantial (up to 37%) underestimation of flow and velocity measurements with the k−t acquisition in the splenic vein, hepatic artery and celiac trunk.

Bollache et al.^18^ examined four k-space ordering schemes for k−t accelerated 4D flow MRI without respiratory control in a flow phantom mimicking the aortic arch, and in the thoracic aorta of volunteers and patients. They found that the k−t accelerated sequence with the out-center-out pattern achieved equivalent quantification of velocity and flow compared to a standard Cartesian navigator-gated sequence, albeit with a lower image quality in the descending aorta. In the phantom, there was a small under-estimation of maximum velocity (−0.8%) and flow (−0.5%) in the portion mimicking the aortic arch, but larger underestimation (−18%) for peak velocity in the coarctation of the descending aorta, with more turbulent and complex flow. The authors hypothesized that the out-center-out pattern provides equivalent quantification in shorter acquisition time because the corners of k-space are acquired first to keep the scan time short, while center k-space is acquired later, allowing the patient to reach a stable respiratory pattern which increases image contrast. We imaged the abdominal vasculature with the same version of the sequence, similar temporal resolution (71 ms) and higher, isotropic spatial resolution (2.5 × 2.5 × 2.5 mm^3^) than used in the thoracic aorta (67.2 ms and 3.4 × 2.3 × 2.6 mm^3^)^18^. We achieved similar absolute mean intersequence differences in peak velocity (observer 1 and 2: 6.9 ± 22.1%/−1.8 ± 8.3%) and flow (6.3 ± 12.9%/2.9 ± 10.5%) in the abdominal aorta above the celiac trunk, as in the Bollache et al. study in the descending thoracic aorta of patients (peak velocity: −5.0 ± 12%; flow: 5.8 ± 6.3%).

Stankovic et al.^19^ compared respiratory gated, k−t accelerated 4D flow MRI acquisitions using a time-interleaved sampling pattern with similar spatial (2 × 2.4 × 2.4 mm^3^) and temporal (41–82 ms) resolutions, and R = 3–8 acceleration factors, to standard respiratory gated 4D flow MRI with GRAPPA = 2 acceleration, in the hepatic circulation of healthy volunteers. For the hepatic vessels, our study observed similar Bland–Altman biases between our R = 5 k−t accelerated free-breathing acquisition and the standard navigator-gated acquisition, but wider limits of agreement than in this previous study. The flow and velocity values in the abdominal circulation measured with both acquisition methods in our study were in agreement with published 4D flow MRI values measured in patients with chronic liver disease with conventional navigator-gated Cartesian^1,3^, navigator-gated radial^10,11^ and spiral^5,7^ 4D flow MRI acquisitions. The decreased image quality and underestimation of peak velocity and flow values with the k−t accelerated acquisition suggest that the k−t out-center-out acquisition with no respiratory control is not suitable for hemodynamic quantification for small arteries and the splenic vein. The arteries are typically smaller in diameter (in patients with liver disease: celiac trunk: 7–8 mm; hepatic artery: 3–4.5 mm^20^; splenic artery: 5–6 mm^20^) than the portal vein (14–15 mm in patients with liver disease^21^) and abdominal aorta (20–30 mm). In the splenic vein, significantly lower vessel conspicuity with the k−t acquisition was associated with (and possibly a cause of) poorer interobserver reproducibility and under-estimation of flow values by 30–37% by both observers. The hepatic artery, splenic artery and vein, are also more tortuous, resulting in complex flow patterns. Stankovic et al.^19^ showed that while controlling for respiratory motion, k−t accelerated acquisitions with R = 3–5 acceleration factors achieved the same image quality and quantitative values as standard 4D flow MRI with GRAPPA = 2 in healthy volunteers, in all hepatic vessels, including small arteries. Thus, respiratory blurring, rather than k−t undersampling, may have a stronger negative impact on image quality and velocity quantification in smaller vessels than larger ones. Veins also have a slower flow compared to arteries, and the under-sampled k−t acquisition may not have been able to resolve smaller differences in venous velocities. Thus, a higher spatial resolution k−t accelerated acquisition combined with respiratory control is needed for optimal imaging of these vessels. Using a respiratory navigator with 60–80% scan efficiency, such as the ReCAR navigator^14^ or the navigator proposed by van Ooij et al.^22^ in combination with the k−t accelerated 4D flow MRI sequence, the 3 -minute free-breathing acquisition would take 4–5 min, still providing time savings compared to the conventional navigator-gated 4D flow MRI acquisition of 9–10 min. The k−t accelerated acquisition as performed in our study can be used to measure flow in the portal vein and supraceliac aorta in patients for whom shorter scan time is needed, as it allows adequate quantification in those vessels.

The liver 4D flow MRI study of Dyvorne et al.^5^ assessed a one-breath-hold, spiral 4D flow acquisition with compressed sensing reconstruction that achieved an acceleration factor R = 6, against a navigator-gated Cartesian acquisition with parallel imaging R = 2. Bland–Altman bias between average velocity and average area measurements in the portal vein with the spiral vs. the Cartesian sequence was close to zero, better than in our sequence comparison. The interobserver repeatability of the spiral sequence in the abdominal vessels was poorer (CV range in hemodynamic parameters: 14.9–21.6%)^7^ compared to the k−t accelerated sequence (CV range: 9.9–13.4%). However, disadvantages of the spiral acquisition are its respiratory control through long breath-hold (22 s), which is not feasible in very sick patients, its lower resolution (2.5 × 2.5 × 5 mm^3^ vs. 2.5 × 2.5 × 2.5 mm^3^ with the k−t accelerated acquisition), which makes measurements in the small vessels less robust, and less efficient offline reconstruction algorithm. Sequence modifications to increase the acceleration factor and provide inline reconstruction can mitigate these drawbacks.

In this small initial series of 7 patients with HVPG measurements, average velocity in the superior mesenteric vein (measured with the k−t accelerated acquisition) correlated with HVPG, which agrees with a previous study showing positive correlation between average and peak velocity in the superior mesenteric vein^7^ and an imaging score predictive of PH^23^. However, this finding was not reproduced in our study with the reference navigator-gated sequence and should be confirmed in a larger study.

This study had a few limitations. First, the sequence comparison was performed in a small number of subjects, and we did not conduct a test–retest repeatability study^7^. Second, due to integration of the 4D flow acquisitions in a multiparametric MRI protocol, it was not feasible to compare the k−t accelerated acquisition with and without respiratory control to the navigator-gated reference sequence, or to examine different k-space ordering patterns of the k−t accelerated sequence, as done in the aorta^18^. As our imaging protocol included DCE-MRI, it was not possible to acquire 4D flow during the arterial or portal venous phase, and thus benefit from gadolinium-enhanced SNR**.** For consistency of protocol, we performed the two acquisitions in the same order, rather than in random order; however, since the hepatobiliary agent used clears the vasculature by 10 min after injection, the sequence acquired first would not have benefited from increased SNR. Third, hemodynamic measurements were made in single planes perpendicular to the vessels of interest, which led to greater interobserver and intersequence bias.

The k−t accelerated acquisition protocol can be improved by image matrix optimization to decrease the 30 min reconstruction time for the k−t accelerated acquisition in the abdomen to 15 min like in the thoracic aorta^18^. Reconstruction time can be shortened further by using GPU computing. Future work will include comparisons of 4D flow MRI acquisitions in the abdomen with different acceleration methods (e.g. spiral with compressed sensing vs. k−t GRAPPA), and of multiple methods of respiratory control (no respiratory control, breath-hold or navigator gating). Comparisons of different motion correction and vessel segmentation strategies in post-processing software is also a worthwhile area of investigation, as is 3D measurement of hemodynamic parameters by automated vessel segmentation^24^ and placement of multiple perpendicular planes along the vessels of interest^25^.

In conclusion, k−t accelerated 4D flow MRI with no respiratory control allows three-times shorter acquisition time, but with underestimation of maximum velocities and flow in the small arteries and the splenic vein compared to conventional navigator-gated 4D flow MRI. Respiratory gating and technical improvements to increase resolution of the k−t accelerated acquisition may be needed for accurate flow quantification in these abdominal vessels.

## Methods

### Image acquisition

This prospective, cross-sectional, single-center study was approved by the IRB at the Icahn School of Medicine at Mount Sinai and complied with the Declaration of Helsinki, good clinical practices, HIPAA and GDPR protections for human subjects. Written informed consent was obtained for all patients. Adult patients with chronic liver disease and suspected portal hypertension (PH), who had invasive measurement of the hepatic venous pressure gradient (HVPG) were referred by our institution’s Hepatology department for inclusion in the study. Patients with prior/concomitant pharmacologic treatment for PH, portal vein thrombosis (as determined based on prior clinical imaging) or contraindications for MRI, were excluded.

All patients fasted for at least 4 h before the MRI. MRI-compatible ECG leads were placed on the patients’ chest before imaging. Patients were imaged on a 1.5 T system (Magnetom Aera, Siemens Healthineers, Erlangen, Germany) with 33 mT/m maximum gradient strength, equipped with a 32-channel spine and 18-channel body matrix coil. 4D flow sequence was acquired as part of a 60 min. multiparametric MRI protocol.

The 4D flow acquisitions were performed with: (1) a Cartesian, respiratory navigator-gated 4D flow prototype sequence with Respiratory Controlled Adaptive k-space Reordering (ReCAR) navigator technology^14^ and (2) a free-breathing Cartesian k−t GRAPPA accelerated 4D flow sequence with an out-center-out k-space sampling pattern previously validated in the thoracic aorta^18^. In the out-center-out k-space sampling pattern, the ky-kz space corners were filled during the first 10 cardiac cycles, followed by center-out filling of k-space, from the central (ky = kz = 0) position to the outer k-space positions^18^.

**Figure 4:**
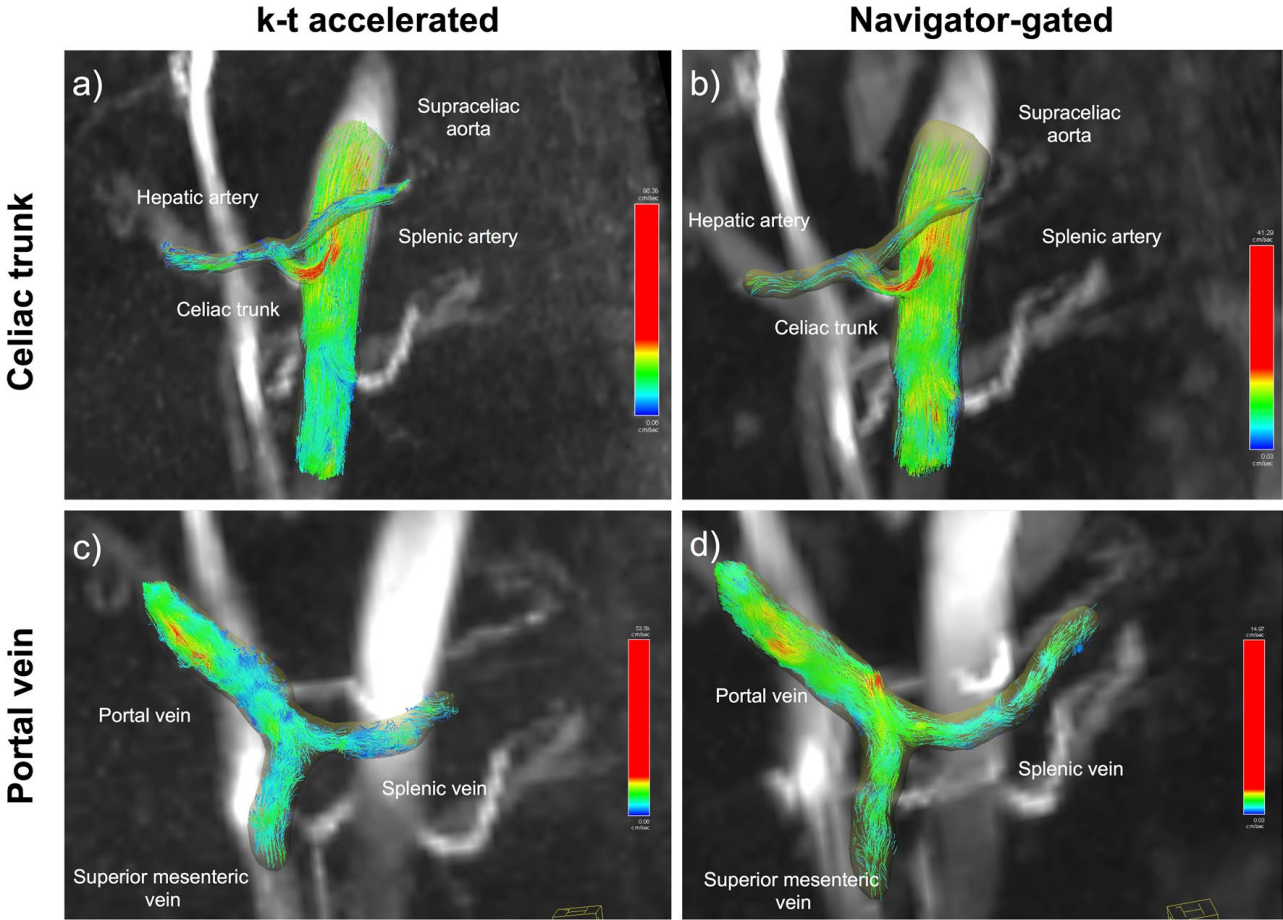
60 year-old male with HCV cirrhosis and no portal hypertension (HVPG = 0 mmHg). Example of celiac trunk (**a**, **b**) and portal vein (**c**, **d**) segmentation with streamlines showing flow from the k−t accelerated (**a**, **c**) and the navigator-gated (**b**, **d**) acquisitions. Vessel depiction was of excellent quality for both acquisitions. (**a**) For the k−t accelerated acquisition, time-averaged velocity and flow in the hepatic artery were 7.3 cm/s and 2.3 ml/s, in the celiac trunk were 21.8 and 9.7 cm/s, and in the splenic artery were 4.8 and 2 cm/s. (**b**) For the navigator-gated acquisition, time-averaged velocity and flow in the hepatic artery were, and 5.9 cm/s and 2.8 ml/s, in the celiac trunk were 14.6 and 6.8 cm/s, and in the splenic artery were 7.2 and 3.1 cm/s. (**c**) With the k−t accelarated acquisition, time-averaged velocity and flow in the portal vein were 5.6 cm/s and 9.6 ml/s, in the superior mesenteric vein were 4.6 and 4.9 cm/s and in the splenic vein were 2.5 and 2.3 cm/s. (**d**) For the navigator-gated acquisition, time-averaged velocity and flow in the portal vein were and 6.0 cm/s and 10.3 ml/s, in the superior mesenteric vein were 5.9 cm/s and 6.7 cm.s, and in the splenic vein were 5.3 cm/s and 4.3 cm/s.

Both coronal-oblique acquisitions covering the abdominal vessels (Fig. 4) with matched parameters (TR/TE/FA: 5.9 ms/3.4 ms/15º, field of view 400 × 400 mm, 24 slices, slice thickness 2.5 mm, matrix size 160 × 160, voxel size (2.5)^3^ mm^3^, 3 k-space lines/cardiac cycle, temporal resolution 71 ms) were obtained 10 min after injection of gadoxetic acid (Eovist/Primovist, Bayer Healthcare Pharmaceuticals). Respiratory-navigator gated Cartesian 4D flow, acquired first and used as reference, followed acquisition parameters previously validated for liver vasculature^5,7,26^, with a GRAPPA acceleration factor R = 2 in the y-direction, prospective ECG gating and respiratory control via two pencil-beam navigators placed on the spleen-lung interface, away from liver parenchyma and vessels, with an acceptance window of ± 8 mm. The k−t accelerated acquisition used a PEAK-GRAPPA spatio-temporal acceleration factor R = 5 in the y- and z-directions, prospective ECG gating, but no respiratory control, as previously demonstrated in the aorta^18^. Both acquisitions used a velocity encoding parameter (VENC) of 60 cm/s, as previously used in 4D flow studies of liver disease^1,4,5,7,8,10,27^. For both 4D flow sequences, the acquisition and reconstruction time was recorded. Inline reconstruction time for the k−t acquisition was 30 min., while the reconstruction time for the navigator-gated acquisition was 2 min. per dataset.

### Image analysis

Two independent observers (O.B., an MR physicist with 5 years’ experience, and D.S., a body radiologist with 6 years’ experience in abdominal imaging) performed vessel segmentation for the respiratory-navigated and k−t accelerated acquisitions. Images were analyzed using prototype software (4D Flow Demonstrator, Siemens Healthineers) for 4D flow data visualization and vessel segmentation using a graph-cut centerline model^28^. The software performed corrections for eddy currents and motion, as well as for phase aliasing, by correcting for pairs of opposite changes in velocity larger than 2xVENC in the velocity–time curves^28,29^. After performing the corrections, the portal, superior mesenteric, splenic and middle hepatic veins, the supraceliac aorta, celiac trunk, hepatic and splenic arteries (Fig. 4), were identified and segmented from a 3D phase-contrast angiogram constructed based on the 4D flow velocity data. Anatomical T_2_-weighted and post-contrast T_1_-weighted imaging was used as a reference for vessel identification. Particle traces were visualized in all vessels to confirm physiological direction of flow, and to identify if a hepatofugal flow pattern was present in the portal vein.

### Qualitative evaluation

Both observers provided qualitative scoring of vessel conspicuity (0, vessel not seen; 1, severely to moderately blurred; 2, mildly blurred; and 3, well delineated) and background noise and artifacts (1, severe; 2, moderate; and 3, minimal/none) for both acquisitions. Figure 1 shows examples of different degrees of vessel conspicuity and background noise and artifacts.

### Quantitative evaluation

The measurement planes were placed in areas of the vessel with high density of particle tracings. Conservation of mass between the portal vein and the mesenteric-splenic vein confluence, and the celiac trunk and the hepatic and splenic arteries were used as an internal consistency check of measurements. Peak through-plane velocity, time-averaged vessel cross-section area, through-plane velocity and flow were measured.

### Statistical analysis

Inter-acquisition and inter-observer agreements on vessel identification were evaluated by Cohen’s kappa, and were considered poor for kappa < 0.2, fair for kappa = 0.21–0.4, moderate for kappa = 0.41–0.6, substantial for kappa = 0.61–0.8, and excellent for kappa > 0.8^30^. Inter-acquisition and inter-observer agreement of measurements was evaluated by coefficient of variation (CV) and Bland–Altman statistics. Inter-observer and inter-acquisition reproducibilities of 4D flow hemodynamic measurements were considered to be excellent if CV < 10%, substantial if CV < 15%, and acceptable if CV < 20%, comparative to 4D flow measured velocity in the aorta^31^. Differences in measurements between the k−t accelerated and navigator-gated acquisition were calculated and expressed as percentage of the navigator-gated measurement. The statistical significance of differences in parameters and quality scores between acquisitions was tested using paired Wilcoxon. Hemodynamic parameters were correlated with HVPG using Spearman and Pearson correlation. Two-sided *p* values < 0.05 were considered significant. Statistical tests were performed using SPSS v.20 (IBM) and MATLAB R2016a (The Mathworks, Natick, MA).

## Data Availability

Anonymized 4D flow MRI datasets with patients’ relevant clinical information will be made available three months after publication, by directly contacting the corresponding author.
